# Assessing the shock state of the lunar highlands: Implications for the petrogenesis and chronology of crustal anorthosites

**DOI:** 10.1038/s41598-017-06134-x

**Published:** 2017-07-19

**Authors:** J. F. Pernet-Fisher, K. H. Joy, D. J. P. Martin, K. L. Donaldson Hanna

**Affiliations:** 1 School of Earth and Environmental Sciences, University of Manchester, Manchester, M13 9PL UK; 2 Atmospheric, Oceanic and Planetary Physics, University of Oxford, Oxford, OX1 3PU UK

## Abstract

Our understanding of the formation and evolution of the primary lunar crust is based on geochemical systematics from the lunar ferroan anorthosite (FAN) suite. Recently, much effort has been made to understand this suite’s petrologic history to constrain the timing of crystallisation and to interpret FAN chemical diversity. We investigate the shock histories of lunar anorthosites by combining Optical Microscope (OM) ‘cold’ cathodoluminescence (CL)-imaging and Fourier Transform Infrared (FTIR) spectroscopy analyses. In the first combined study of its kind, this study demonstrates that over ~4.5 Ga of impact processing, plagioclase is on average weakly shocked (<15 GPa) and examples of high shock states (>30 GPa; maskelynite) are uncommon. To investigate how plagioclase trace-element systematics are affected by moderate to weak shock (~5 to 30 GPa) we couple REE+Y abundances with FTIR analyses for FAN clasts from lunar meteorite Northwest Africa (NWA) 2995. We observe weak correlations between plagioclase shock state and some REE+Y systematics (*e.g*., La/Y and Sm/Nd ratios). This observation could prove significant to our understanding of how crystallisation ages are evaluated (*e.g*., plagioclase-whole rock Sm-Nd isochrons) and for what trace-elements can be used to differentiate between lunar lithologies and assess magma source compositional differences.

## Introduction

The lunar surface has been subjected to periods of intense meteorite bombardment over geologic time (*e.g.*, refs 1, 2.) Understanding how lunar lithologies have been modified during impact events represents an important ‘first-order’ question that has implications for our understanding of regolith formation and impact structures on all rocky Solar System bodies^3^. The majority of samples from the lunar surface available to us for study have to some degree been affected by impact modification; ranging from rapid shock pulses resulting in the structural deformation of minerals (*i.e*., planar deformation effects^4, 5^); to longer duration thermal heating effects (*i.e*., chemical re-equilibration within and between mineral assemblages^6, 7^). Understanding the effects of these processes is of critical importance for lunar highland derived lithologies, as these samples are vital for understanding the earliest records of lunar crustal history^8, 9^.

The primary lunar crust is thought to consist of predominantly plagioclase-rich units (ferroan anorthosite; FAN) consisting of >90% by mode Ca-rich plagioclase with a restricted An % range (~96 to ~98), and minor quantities of olivine and/or pyroxene phases with relatively ferroan compositions (Mg # ~40 to ~70 in orthopyroxene and olivine)^10^. However, only a few anorthosites have been recovered as hand specimens (2.8% of >10 mm sized samples recovered from the Moon^11^). Most anorthositic material is commonly sampled as small (<10 mm sized) rock fragments within the lunar soil, or clasts within regolith and fragmental breccias. It is the study of these small clasts that much of our understanding of the Moon’s earliest formed crust is based (*e.g*., refs 7, 12–14). Plagioclase, unlike olivine and pyroxene, is readily affected by shock, making it a useful mineral to investigate shock effects^15–18^. However, the extent to which these small fragments are representative of their parent bedrock lithology has recently been questioned^19^; furthermore, few studies have systematically deconvoluted the often-complex impact modification history that individual clasts have experienced.

Despite these potential complexities, plagioclase has the potential to record important physical and chemical magmatic processes (*e.g*., magma mixing, mantle source heterogeneity) as its structure incorporates several elements whose ionic radii are too large to fit into earlier-crystallising ferromagnesian minerals; particularly large ion lithophile elements (LILE; *i.e*., Sr, K, Rb, Ba) and the Light Rare Earth Elements (LREE)^20, 21^. To date, few studies have directly characterised how trace-element abundances within high-Ca plagioclase vary with degree of shock; those existing studies have focused predominantly on the behaviour of compatible minor- and trace-elements such as K and Sr^7, 22–25^.

In this study, we characterise the range of shock damage recorded by anorthitic plagioclase in lunar feldspathic samples, and calculate the relative extent of maximum shock pressures experienced by individual plagioclase crystals. We also present REE+Y data for a number of shocked plagioclase crystals to directly investigate the extent to which shock damage is able to modify incompatible trace-element systematics. Understanding this relationship is important for studies that wish to constrain the composition of the parental melts to the highland lithologies, but also for studies that wish to use chronometers such as the Sm-Nd or Ar-Ar isotope systems to estimate the crystallisation ages or identify isotopic resetting events.

## Samples

**Figure 1:**
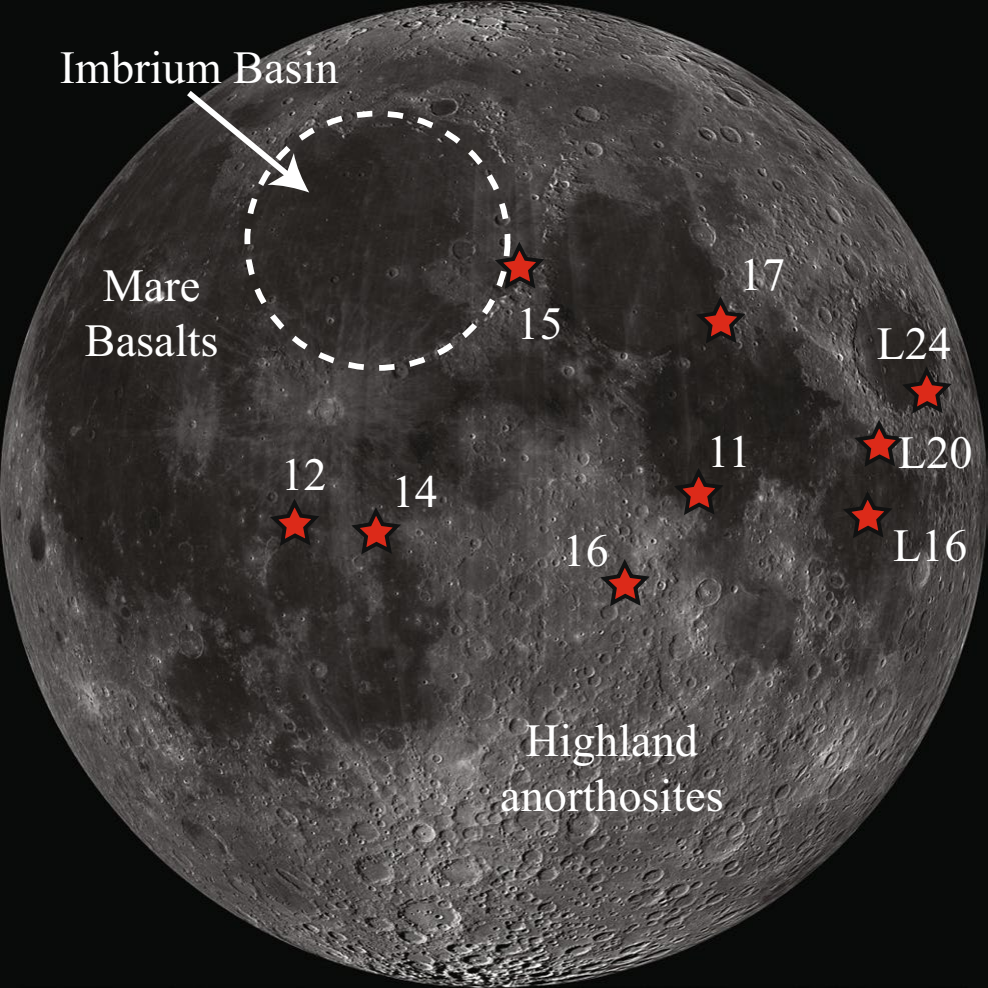
Sample collection context map. Lunar Reconnaissance Orbiter (LRO) Wide Angle Camera (WAC) mosaic (Image from the LROC PDS archive: NASA/GSFC/Arizona State University) of the lunar nearside, showing dark mare basalts and the higher albedo highland rocks. Stars represent the location of the Apollo and Luna (L) mission landing sites. Dashed circle marks the estimated limit of the rim of the Imbrium basin.

Here we investigate a suite of polished thin-sections from the Apollo FAN hand sample collection (15415, 60015, 60135, 62236, 62275) and Apollo 16 regolith breccias that contain a range of FAN lithic clasts and mineral fragments (60016, 61175, 61135). The rocks collected during the Apollo 16 mission represent the only direct sampling of the central near-side lunar highlands, a region which is heavily cratered (Fig. 1). Lithologies sampled during this mission include magmatic rocks from the Mg-Suite (Mg-rich norite/gabbronorites/troctolites), the High Alkali Suite (HAS), and primary crustal rocks from the ferroan anorthosite (FAN) suite in addition to a wide range of impact generated lithologies. The Apollo 16 breccias are fused regolith samples that contain a mixture of all these lithologies present as small mineral fragments (<100 μm) to large (~5 mm) clasts, in addition to containing glass spherules, impact melts, and agglutinates^26–28^. Apollo FAN hand specimens and FAN-derived clasts within breccias are likely related to the excavation of crustal lithologies by the Imbrium basin-forming event^27^. Based on previous analyses^11^ all Apollo 15 and 16 hand samples investigated here have been classified as chemically pristine, meaning that they do not contain added chemical components from impactor material such as elevated siderophile-element concentrations. In addition to the Apollo samples, we also investigated clasts from lunar meteorite Northwest Africa (NWA) 2995. This sample is classified as a lunar feldspathic (fragmental) breccia, containing a minor mafic component^29^. In order to investigate relationships between shock state and chemistry we focus specifically on NWA 2995, as by the lithologic nature of this sample it is most likely to display a varied shock history. Furthermore, by focusing on one sample, this limits the effects of intrinsic heterogeneities (due factors such as mantle source variations) that may obscure such relationships.

## Results

### Mineral chemistry

**Figure 2:**
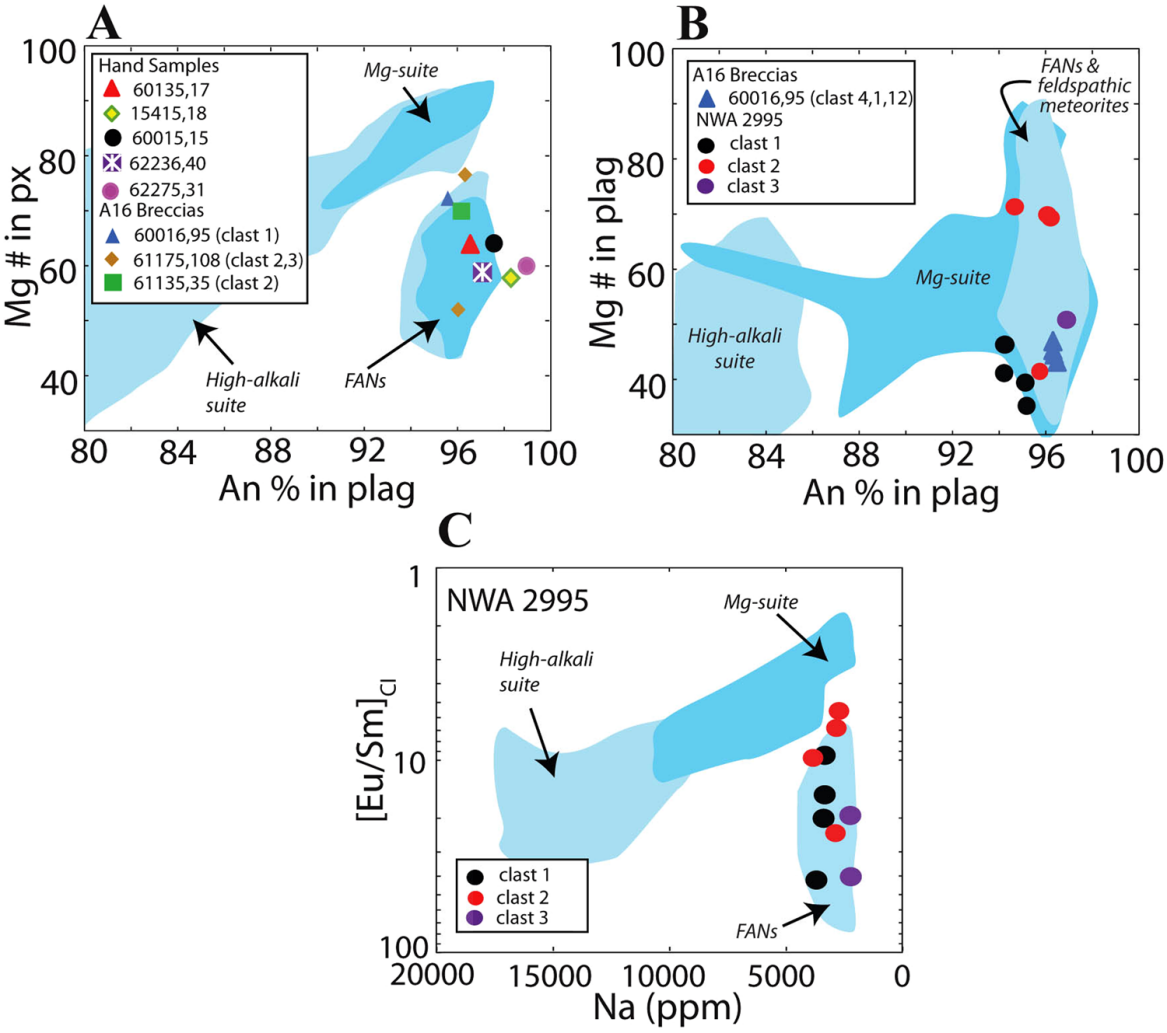
(**A**) Average An % (100*Ca/[Ca+Na+K]) in plagioclase vs. Mg # (100*Mg/[Mg+Fe]) in pyroxene for FAN hand samples (mineral chemistry data from^32^). Background fields mafic mineral Mg # and plagioclase An % for main Apollo highland rock suites are taken from Yamaguchi *et al*.^65^. (**B**) Average An % vs. Mg # in plagioclase for clasts and mineral fragments within Apollo 16 breccias. (**C**) [Eu/Sm]_CI_ vs. Na ppm in plagioclase for clasts within lunar meteorite NWA 2995. Background fields for **B** and **C** have been made using data reported in refs 7, 8, 31, 66, 67.

Plagioclase from the FANs are characterised by distinct mineral chemistry with respect to other highland lithologies: they have a restricted range of plagioclase calcium content (An % ~96 to ~98), while displaying relatively Fe-rich (*e.g*., Mg # ~40 to 70 in pyroxene) mafic phases relative to other lunar lithologies (Fig. 2A; 10). Averages for plagioclase An % for all plagioclase crystals in hand specimen and breccia clasts investigated in this study fall within this range (94.8 to 97.1 An % for Apollo samples and 95.0 to 97.2 An % for NWA 2995; Fig. 2). All individual measured plagioclase compositions for the Apollo samples are presented in the SOM (Table S1), and plagioclase compositions for NWA 2995 are presented in Table 1. Samples 60016,95 and NWA 2995 contain numerous large (>250 μm) plagioclase fragments with no associated mafic phases. In order to constrain the parent lithologies of these fragments, we have plotted these clasts' An % against plagioclase Mg #. Whereas the plagioclase fragments from the Apollo 16 breccias and NWA 2995 are within range of reported FAN values (Fig. 2B), there is overlap with other lunar lithologies, in particular with the Mg-suite. Using the lunar rock suite classification scheme derived from [Eu/Sm]_CI_ vs. Na ppm abundance^30^, the majority of plagioclase fragments from NWA 2995 fall within the FAN field (Fig. 2C). A petrologic summary (mineral modes and sample mass/clast size) is presented in Table 2.

**Table 1:** NWA 2995 plagioclase major-elements as measured by EMP) and trace-elements (by SIMS) For SIMS data % Relative Standard deviation (%RSD) is also shown. Reference images of these clasts are shown in Fig. S7.

	NWA 2995 Clast 1								NWA 2995 Clast 2								NWA 2995 Clast 3			
	Plag 1	%RSD	Plag 2	%RSD	Plag 3	%RSD	Plag 4	%RSD	Plag 7	%RSD	Plag 8	%RSD	Plag 9	% RSD	Plag 10	% RSD	Plag 11	%RSD	Plag 12	%RSD
EMPA (wt%)																				
SiO_2_	44.6		44.6		42.7		44.4		43.3		43.9		43.8		43.4		43.2		43.5	
Al_2_O_3_	35.3		35.3		34.3		35.6		35.8		35.9		36.4		35.7		34.9		34.7	
MgO	0.13		0.13		0.10		0.06		0.08		0.10		0.07		0.08		0.03		0.04	
CaO	19.4		19.4		19.1		19.6		19.5		19.1		19.6		19.1		20.0		20.0	
FeO	0.34		0.34		0.26		0.26		0.10		0.09		0.10		0.21		0.11		0.13	
Na_2_O	0.60		0.60		0.46		0.50		0.37		0.52		0.38		0.39		0.30		0.30	
K_2_O	0.02		0.02		0.06		0.01		0.03		0.06		0.02		0.06		0.02		0.02	
Total	100.4		100.4		96.9		100.4		99.2		99.7		100.3		99.0		98.6		98.7	
SIMS (ppm)																				
Ti	100.3	1	294.6	1	139.0	1	88.4	1	201.0	1	83.8	1	243.1	1	73.1	1	230.4	1	89.0	1
Sr	144.3	0.4	268.1	0.3	200.1	0.3	197.2	0.3	215.0	0.3	195.4	0.3	220.7	0.3	234.2	0.3	171.3	0.3	172.1	0.4
Y	0.78	4	1.59	3	0.47	4	0.35	5	1.55	3	1.44	3	1.66	2	0.41	5	0.85	3	0.27	7
Ba	18.60	2	35.46	1	16.13	2	13.93	2	64.68	1	34.83	1	74.85	1	23.63	1	10.34	2	8.83	2
La	0.54	6	1.26	4	0.62	6	0.37	7	2.48	3	1.00	5	3.31	3	0.43	7	0.21	10	0.20	11
Ce	1.20	5	3.06	3	1.34	4	0.86	5	5.38	2	2.41	3	6.96	2	0.87	5	0.48	7	0.42	9
Pr	0.14	11	0.43	7	0.13	11	0.11	13	0.63	6	0.32	8	0.73	5	0.11	13	0.06	17	0.05	20
Nd	0.73	15	2.09	9	0.72	14	0.45	18	3.10	8	1.41	11	3.28	7	0.54	17	0.39	19	0.23	28
Sm	0.14	29	0.54	15	0.15	27	0.06	42	0.57	16	0.32	19	0.52	14	0.12	30	0.12	31	0.05	50
Eu	0.78	7	1.94	5	1.13	6	0.96	6	1.19	6	1.18	5	1.33	5	1.10	6	0.85	7	0.82	8
An %	94.6		94.6		95.5		95.6		96.5		95.0		96.5		96.1		97.2		97.2	
Ab %	5.3		5.3		4.1		4.4		3.3		4.6		3.4		3.5		2.6		2.6	
Or %	0.1		0.1		0.4		0.1		0.2		0.3		0.1		0.4		0.1		0.1	
Mg #	40.9		40.9		40.2		29.1		57.4		66.0		54.4		39.6		29.6		33.2	
GPa^*^	10		7		8		11		14		9		9		3		12		5	

*See SOM for details of calculation. ±3 GPa (1σ) for all samples.

**Table 2:** Estimated shock pressures for highland samples in addition to clast size and plagioclase mod. %.

	Pressure Estimates (GPa)		Ar-Ar plateau age^3^		Sm-Nd		Plag mod. %	Sample mass/clast size^4^
	FTIR method pressure range (±3 GPa)^1^	Rubin *et al*.^4^ shock scheme			age^3^			
			(Ga)	(2σ)	(Ga)	(2σ)		
**Apollo FAN Hand samples**								
60015,124			3.6	±0.8			98	50 g
‘clast’	0 (n = 4)	S1 (0–5)^2^						
‘matrix’	8 to 21 (n = 3)	S2 (5–10)						
15415,18	0 (n = 7)	S1 to S2 (0–10)	4	±0.2				269 g
60135,17	3 to 12 (n = 5)	S2 to S3 (5–15)					95	285 g
62236,40	0 to 6 (n = 9)	S2 to S3 (5–15)	3.9	±0.1	4.29	±0.09	91	9.1 g
62275,31	0 to 12 (n = 18)	S1 to S2 (0–10)					93	443 g
60025			4.2	±0.1	4.37	±0.12	98	1836 g
67215			3.9	±0.1	4.41	±0.13	95	273 g
**Apollo 16 Breccias**								
60016,95			3.8	±0.1				
*clast 4*	0 (n = 4)	S1 (0–5 GPa)					single crystal	0.2 cm
*clast 1*	0 to 15 (n = 5)	S1/S2 (0–10)					disaggregated plag.	0.5 cm
*clast 11*	0 (n = 3)	S1 (0–5)					single crystal	0.3 cm
*clast 12*	0 (n = 3)	S1/S2 (0–10)					single crystal	0.05 cm
61175,108								
*clast 2*	0 (n = 4)	S1 (0–5)					98	1.0 mm
*clast 3*	0 to 40 (n = 4)	S4/S5 (10–60)					96	1.0 mm
61135,36			3.9	±0.1			98	0.75 mm
*clast 2*	0 to 3 (n = 5)	S1 (0–5)						
60035			4.1	±0.1				
66075			3.9	±0.1				
**Meteorite anorthosite clasts**								
NWA 2995								
*clast 1*	0 to 26	N/A					single crystal	100 μm
*clast 2*	7 to 18	N/A					single crystal	150 μm
*clast 3*	15 to 23	N/A					single crystal	100 μm

^1^All FTIR derived pressures have ± 3 GPa uncertainties (1σ) – see SOM methods. ^2^See Table S3 for summary of this classification scheme. ^3^Ages complied from^32^ and references therein. ^4^Clast size measured along the longest axis.

**Figure 3:**
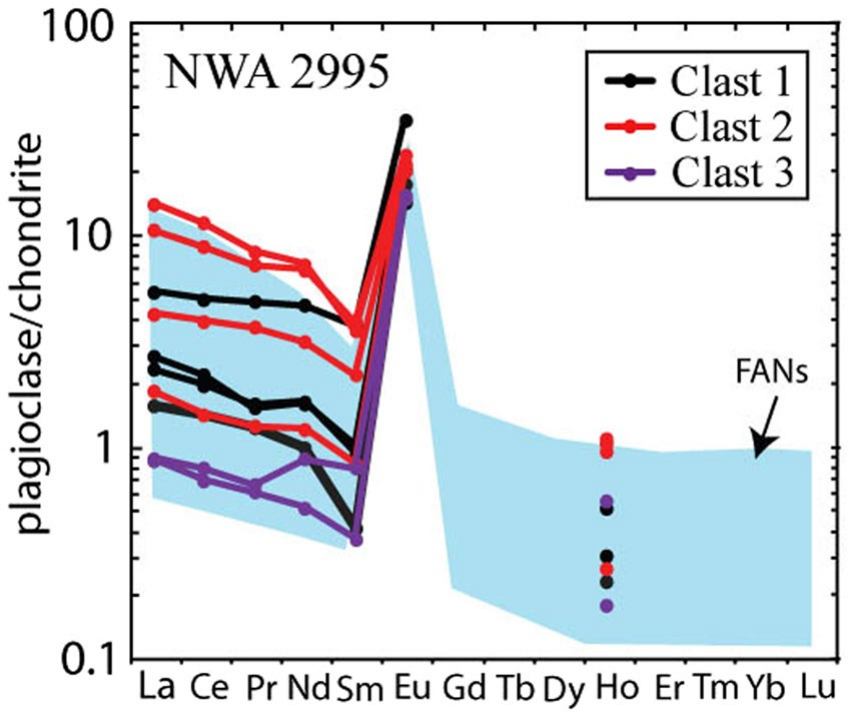
Chondrite normalised REE abundances for plagioclase mineral fragments within NWA 2995, where the chondrite normalisation values are after^61^. Background field for Apollo FAN samples using data collected by ref. 8.

Select plagioclase minor- and trace-element systematics, including REE+Y abundances were analysed for 3 large (>500 μm) plagioclase fragments in NWA 2995 (abundances reported in Table 2). Chondrite normalised REE values analysed here fall within the range reported for Apollo FAN samples (Fig. 3) ^8, 31^. Plagioclase REE profiles display weak to moderate LREE enrichments ([La/Sm]_CI_ 1.1 to 4.0) and strong positive Eu-anomalies ([Eu/Sm]_CI_ 5.6 to 42.0). Most HREE were below detection limits.

### Petrographic shock deformation

A number of anorthosites investigated here are commonly described as being ‘cataclastic’ (*e.g*., ref. 32) indicating that they have been extensively fractured and deformed as a result of impact modification. The characterisation of mineral shock state has traditionally been classified using polished thin-sections^4, 33, 34^. This classification scheme, divided into 6 shock states, is based on the behaviour of plagioclase viewed under crossed-polarized light (the characteristic optical properties and equivalent shock pressures are summarised in Table S2). Plagioclase within Apollo FAN hand samples using this scheme range from S1 (0 to 5 GPa) to S3 (5 to 15 GPa) and Apollo 16 breccias range from S1 to S4/S5 (10 to 60 GPa), reflecting the identification of one maskelynite fragment (the high shock >~30 GPa plagioclase pseudomorph) in sample 61175,108. Only thick-sections were available for NWA 2995; as such, shock states using this scheme could not be used. Indeed, as this method relies on polished thin-sections to estimate shock states, the advantage of using FTIR and CL-imaging is that shock state can be characterised for samples where only thick-sections or polished chips are available.

### Plagioclase FTIR spectra

**Figure 4:**
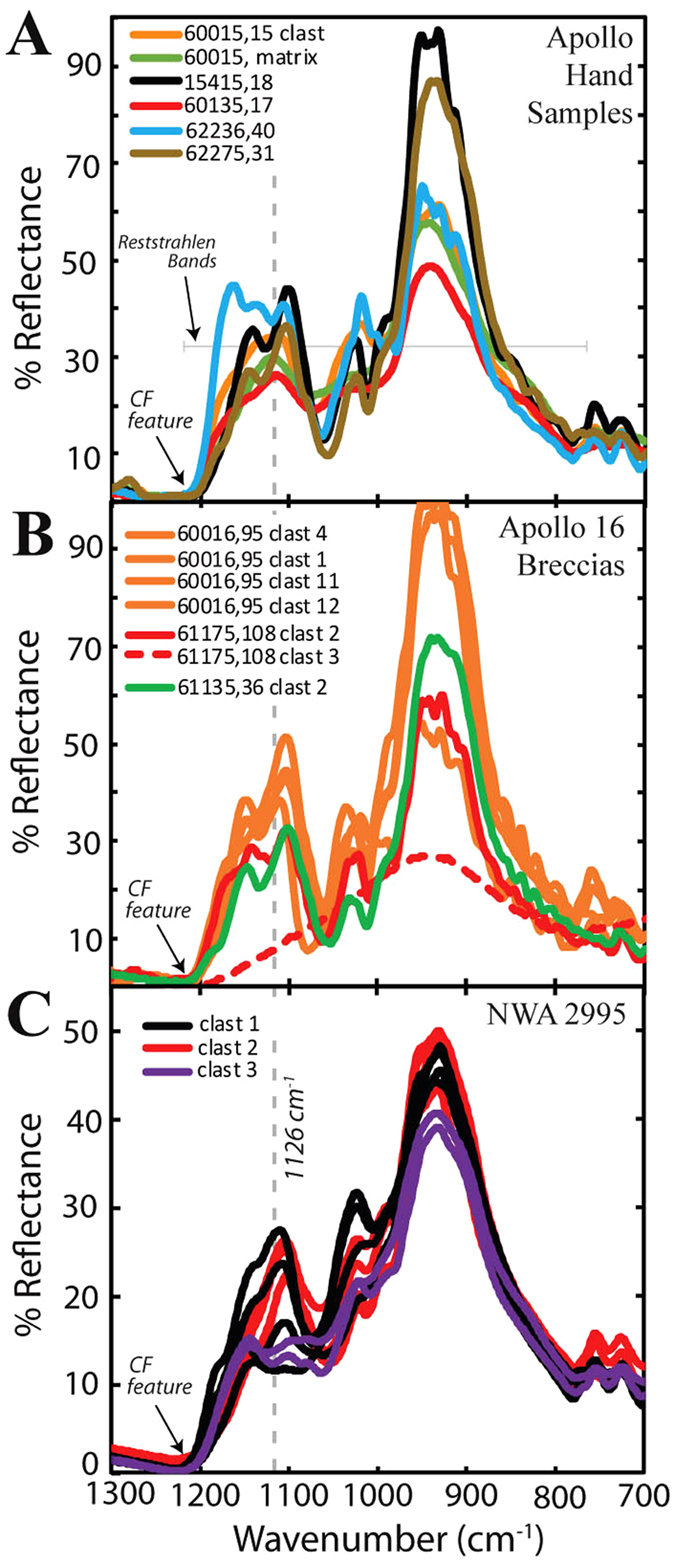
FTIR reflectance spectra (%) for plagioclase within (**A**) FAN hand-samples, (**B**) FAN clasts within Apollo 16 Breccias, and C. lunar meteorite NWA 2995. Uncertainties on FTIR measurements based on repeat analyses are smaller than the line thickness.

The mid-infrared (~3500 to 650 cm^−1^ or ~3 to 15 μm) region of the electromagnetic spectrum (3–15 µm) is sensitive to the mineral chemistry and crystal structure of silicate minerals. The FTIR reflectance spectra of plagioclase in all the samples investigated are consistent with reported high-Ca plagioclase^5, 35, 36^. Figure 4 plots FTIR reflectance spectra (%) between 700 and 1300 cm^−1^. Within this spectral range, Si-O stretching and bending vibrations in the crystal lattice result in a number of reflectance features, collectively known as the Reststrahlen bands. For coarse-particulate high Ca-plagioclase, two main reflectance features are observed with maxima at ~950 cm^−1^ and 1150 cm^−1^. The strengths and absolute positions of these bands are controlled by a combination of crystal structure (*e.g*. crystal orientation, see SOM) and mineral chemistry^36^. All silicate minerals also display a reflectance minimum known as the Christiansen feature (CF) which is indicative of the bulk composition of the sample. The CF position of the plagioclase investigated here ranges from 1212 to 1246 cm^−1^, consistent with reported high-Ca plagioclase^5, 18, 37, 38^. Maskelynite has been observed in only one clast, from Apollo 16 breccia sample 61175,108 (Clast 3) displaying a distinct FTIR spectrum relative to ‘unshocked’ plagioclase (red stippled line; Fig. 4B). The maskelynite FTIR spectrum is characterised by a single broad reflectance peak (Full-width half-maximum value are 243 cm^−1^ and 120 cm^−1^ for maskelynite and crystalline plagioclase respectively) with a maximum at ~930 cm^−1^, a CF position of 1242 cm^−1^ (within range of plagioclase^39^), and typically a lower maximum % reflectance (~30%) relative to plagioclase, consistent with reported maskelynite^35^. This occurrence of maskelynite is within the core of a plagioclase, characterised by its ‘unshocked’ plagioclase FTIR profile (Fig. S9 for photomicrographs under plain and polarised light; maskelynite core is isotropic under crossed polarised light). It’s textural appearance suggests that this is likely to be maskelynite rather than amorphous glass, furthermore, it is spectrally distinct from amorphous impact melt-glass^18^. Together, the plagioclase spectra reported in this study display a range of intermediate spectra, from diagnostic ‘crystalline’ anorthite reststrahlen bands in the 900 to 1200 cm^−1^ region (characteristic of ‘crystalline’ anorthite) to a single reflectance band near ~930 cm^−1^ characteristic of maskelynite, reflecting intermediate shock states^5, 18, 39^. Full resolution laboratory spectra for all plagioclase analyses are reported in Table S1. The FTIR analyses for sample NWA 2995 have been conducted on the same spots as the SIMS trace-element analyses.

### CL imaging

**Figure 5:**
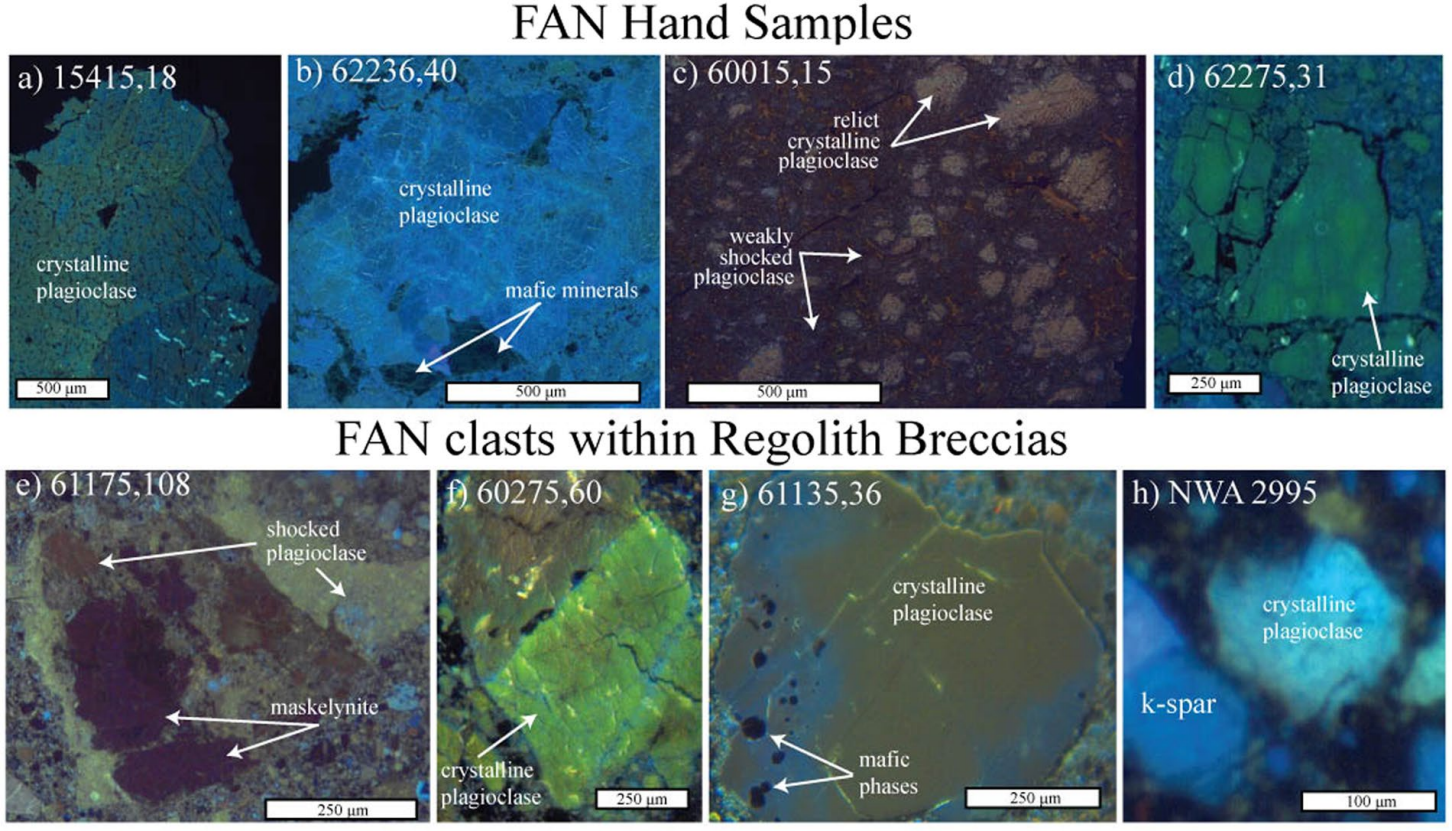
Representative CL-images for both Apollo FAN hand samples (**A**–**D**) and regolith clasts (**E**–**H**). Plagioclase displaying blue/green/orange CL emissions are indicative of plagioclase feldspar. In some cases, CL emission zoning is observed (**G**). Dark inky-blue emissions are indicative of high shock states >~15 Ga (**E**).

All plagioclase crystals for which FTIR spectra were collected were also imaged by OM-CL. Figure 5A–H displays representative OM CL-images together with optical images of the FANs investigated in this study (a full catalogue of CL images for each sample/clast from both Apollo and lunar meteorites investigated in this study is presented within the SOM; Figs S6 and S7). Consistent with previous CL-imaging investigations of plagioclase^40–42^, the visible luminescence of plagioclase ranges from blue (equivalent to wavelengths at ~450 nm; Fig. 5A) to yellow/orange (~560 nm; Fig. 5B,C,F–H). FAN clasts within the Apollo 16 breccias (*e.g.*, 61175 clast 3, Fig. 5D) display zoning between blue ‘rims’ and orange ‘cores’ (such zoning effects have also previously been reported in lunar plagioclase)^30^. However, we observe no relationship between CL-zoning and chemical zoning within the grain (Fig. S5). The maskelynite observed within 61175 is characterised by dark CL emissions (Fig. 5E).

## Discussion

### Characterising shock effects and pressures using CL and FTIR techniques

The visible-light colour CL emission of plagioclase has been interpreted by some to reflect a combination of the extent of ordering within the crystal lattice and the abundance of trace- and minor-elements such as Mn (green/yellow), Fe (red), and Ti (blue)^40, 41^. Indeed, such studies illustrated that compositionally similar shocked plagioclase display systematic changes in CL emissions resulting in a change of colour from green/yellow to orange to blue with increasing shock damage^40, 41^. By coupling FTIR shock pressure analyses with the CL images, for the first time, we are able to test if CL emission change does change as a function of shock pressure.

In order to estimate the maximum degree of shock experienced by a plagioclase of interest using FTIR spectra, we follow a similar approach as Johnson *et al*.^5^. This approach is based on the observation that FTIR reflectance spectra changes systematically with increasing peak shock pressure. In contrast to the polished thin- and thick-sections investigated in this study, Johnson *et al*.^5^ reported bulk spectra for coarse (~1 to 2 mm) high-Ca plagioclase chips. To investigate potential spectral differences between spot analyses of polished sections and ‘bulk’ chips, we analysed both crushed chips (~1 to 2 mm in size, comparable to those reported by Johnson *et al*.^5^) and polished thin-sections of our own reference terrestrial materials (Miyake-Jima anorthite crystals and Stillwater anorthosite, see SOM for details). Despite absolute differences in %R, the relative band positions of both polished sections and chips have overlapping band ratios (Fig. S4), indicating the two methods provide comparable results.

It is important to note that not all changes in the spectral profile of plagioclase are related to shock damage effects. Mineral structural variations, such as crystal orientation, can also have a significant effect on spectral profiles. The well-formed tabular nature of the Miyake-Jima plagioclase crystal used in this study enable an analysis of FTIR spectral variations with crystal orientation planes (“001”, “110”, “101”; Fig. S1). Recognising such effects enable a better understanding of how shock modifies plagioclase FTIR spectra.

In general, the strength of a number of plagioclase spectral features (such as the 790 cm^−1^ to 900 cm^−1^ and 1075 cm^−1^ to 1150 cm^−1^ reststrahlen bands, respectively) have been shown to systematically decrease with increasing shock pressure^5^. Such relationships can then be used for estimating shock pressures for ‘unknown’ plagioclase crystals of interest. Here we use the decrease of reflectance at 1126 cm^−1^ (calculated as the band depth at 1126 cm^−1^ using a continuum removed spectra with tie points at 1075 and 1200 cm^−1^) with increasing shock, based on the relationship defined by experimentally shocked plagioclase to index shock pressure history (Fig. S3). We specifically use this band feature to minimise the effect of spectral changes not related to shock (i.e., crystal orientation), factors not considered by^5^. The result of such ‘non-shock’ factors combined with the external reproducibility (based on repeat measurements of a single crystal faces of Miyake-Jima during the analytical campaign) has resulted in typical errors of ±3 GPa in the estimated pressure reported here. (full details of the method used here including uncertainty estimation are presented in the SOM). Multiple FTIR spots were analysed per plagioclase grain; due to the heterogenous nature of shock on the sub-mm scale, each clast resulted in a range of shock pressures. This range, based on the FTIR analyses, are presented in Table 2 and described below, alongside the Stöffler *et al*.^34^ and Rubin *et al*.^4^ scheme for comparison.

The Apollo samples have undergone estimated shock pressures ranging from unshocked (<5 GPa; equivalent to shock state S1), to very weakly shocked (<12 GPa; equivalent to shock state S2) in FAN hand samples and weakly shocked (15 ± 3 GPa; equivalent to shock state S3) in the breccia clasts (with the exception of the one maskelynite crystal found within clast 3 in breccia sample 61175, which yields a pressure of 40 ± 3 GPa; equivalent to shock state S5; strongly shocked). Plagioclase-bearing clasts within meteorite breccia NWA 2995 have undergone a range of shock pressures from unshocked (<5 GPa; equivalent to shock state S1) to moderately shocked (26 ± 3 GPa; equivalent to shock state S4).

We find no clear relationship between the low to moderate shock indices determined by FTIR and the green/light-blue/orange colour emissions of plagioclase from CL imaging. This range of CL emissions is found in samples that are characterised as unshocked (<5 GPa) by optical and FTIR methods. The lack of such a relationship could reflect small structural changes in plagioclase that are undetectable by optical microscopy and FTIR methods. However, it is likely that the change in plagioclase CL emissions are dominated by chemical variations (*e.g.*, ref. 41) and, for the most part, suggests this is not an effective method for characterising shock <~30 GPa. Only dark inky-blue CL emission are able to distinguish high shock states (i.e., maskelynite; Fig. 5C,E) or amorphous impact glass^18^ relative to crystalline plagioclase.

### Incompatible-element variations in plagioclase with shock

Strong chemical modification of Apollo and lunar meteorite anorthosite plagioclase can occur as the result of highly localised diffusional mechanics during shock-induced thermal metamorphism of the lunar highlands (particularly when plagioclase is located next to a Mg or Fe-rich mineral phase^6, 7^). Measured Mg abundances of plagioclase in NWA 2995 are elevated with respect to reported Apollo 16 FAN samples (>500 ppm Mg in NWA 2995 vs. <500 ppm Mg in the Apollo 16 FANs). However, we find no correlation between the relatively small range of estimated maximum shock pressures experienced by individual plagioclase crystals and their Mg contents (Table 1). The elevated NWA 2995 plagioclase Mg abundances with respect to the FAN plagioclase may reflect either a significant difference in parent melt composition (*e.g*., ref. 14), or subsolidus chemical processing during more prolonged thermal metamorphic events^6^ that are not related to lattice damage caused by short-time interval (<1 minute^33^) impact shock waves. For example, if such a thermal event occurred after the formation of the parent regolith (whereby FAN clasts and mineral fragments are more likely to be in contact with chemically different lithologies such as troctolites or basalts), any prior relationships between Mg and Fe abundance and impact shock pressures would be masked. Thus, careful examination of the petrological context of FAN clasts/fragments within their host regolith breccias must be considered when interpreting mineral chemistry.

**Figure 6:**
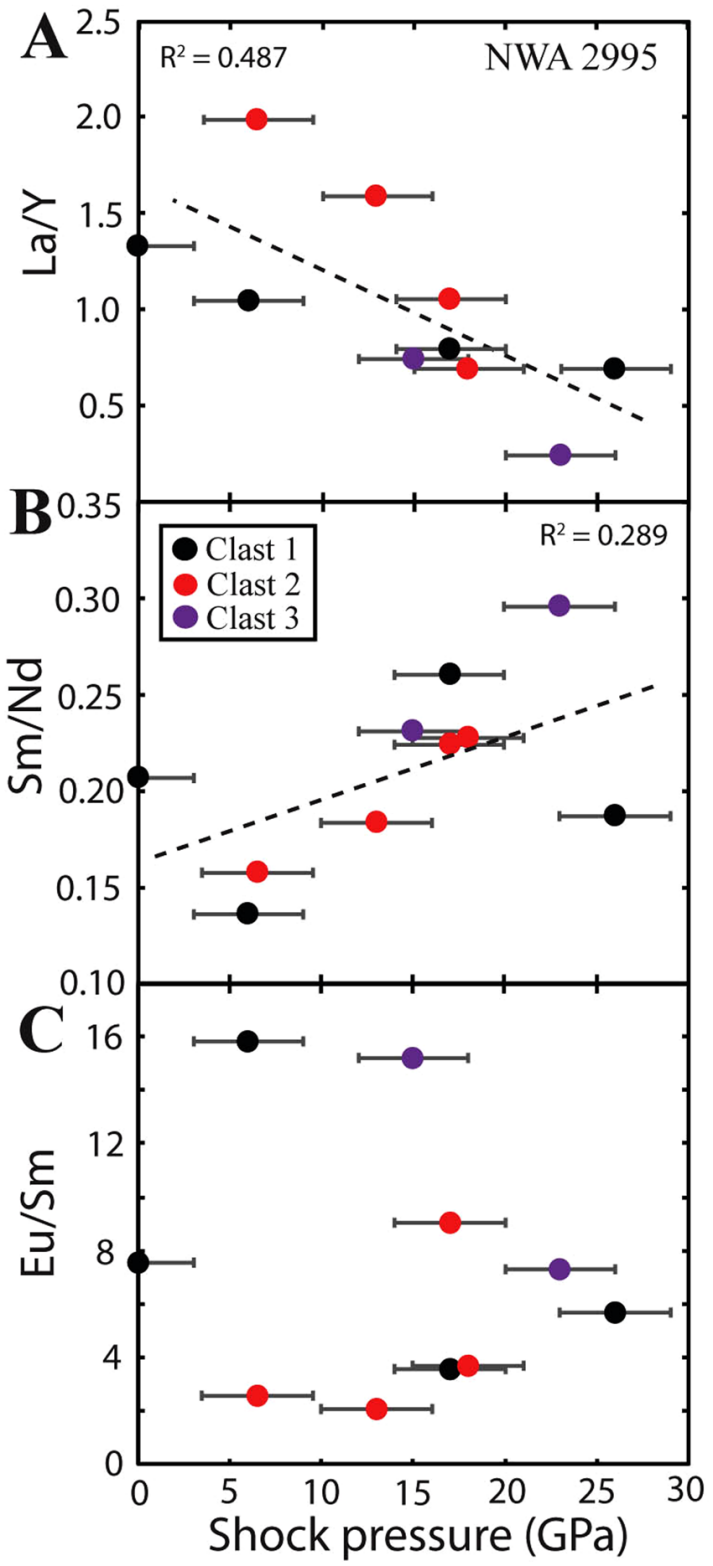
(**A**) La/Y vs. calculated plagioclase peak shock pressure, (**B**) Sm/Nd vs. calculated plagioclase shock pressure (GPa). (**C**) Eu/Sm vs. plagioclase shock pressure (GPa) for FAN fragments within NWA 2995. Best fit lines are plotted for all data points. A weak correlation is observed between Sm/Nd or La/Y and shock states. Error bars represent 1σ (see SOM for details).

Subsolidus re-equilibration between plagioclase and mafic phases has also been suggested to alter mineral REE systematics, particularly following lithology mixing during the formation of regolith breccias and during prolonged thermal metamorphism^8^. The observation of a lack of major-element zoning generally observed for FAN samples, together with lower MgO and FeO abundances relative to what is expected for such An contents^31^, indicates that these samples underwent prolonged periods of thermal metamorphism. The FAN clasts analysed within sample NWA 2995 display weak correlations between some incompatible REE+Y ratios (i.e., ratios not controlled by fractional crystallisation such as La/Y (0.487 r^2^; Fig. 6A or Sm/Nd (0.289 r^2^; Fig. 6B) and shock state <25 GPa in NWA 2995. Typically La/Y are used as a measure of the overall slope of chondrite normalised REE patterns, and Sm/Nd is used as a measure of LREE enrichment and acts as a proxy for the ^143^Sm/^144^Nd ratio used in Nd-isotope radiometric age dating. No correlations are observed between compatible elements and ratios that are controlled by fractional crystallisation (such as Eu/Sm; 0.031 r^2^) and shock state (Fig. 6C). Ratios such as Eu/Sm have recently been used by several studies in order to classify subsets of lunar anorthosites^30^. This suggests that regardless of the weak to moderate shock state typically displayed by lunar highlands plagioclase, robust geochemical information about the original mineral composition can be recovered by analysing the REE+Y, even if some chemical systematics (such as Fe, Mg) have been affected. This is significant, as the robust characterisation of sub-sets within the lunar anorthosite suite has important implications for assessing the composition of the lunar magma ocean, and in turn for testing crustal formation models^8, 14^. Despite this, the low r^2^ seen in plots of La/Y and Sm/Nd and shock state indicate a potential relationship between some trace-element systematics and shock, clearly highlighting the need to take the shock state of samples into consideration when assessing trace-element systematics.

### Implications for geochemical chronometers

The ^147^Sm–^143^Nd isotope system is considered to be one of the most robust chronometers to impact shock modification^25, 43^. However, some mineral open system behaviour (*i.e*., inter- and intra-mineral element diffusion) in the Sm–Nd isotope system has been proposed (*e.g*., refs 44, 45), in some cases suggesting that the youngest reported Sm-Nd ages (~4.2 Ga) could reflect partial re-equilibration ages rather than crystallisation ages (see discussion in refs 46, 47). In particular, Norman *et al*.^45^ noted that ^147^Sm–^143^Nd isochrons that include a plagioclase mineral fraction (such as FAN sample 67215) yielded more scatter than isochrons that only include pyroxene-whole rock fractions, indicating that some plagioclase disturbance in the Sm-Nd isotope system has occurred. Norman *et al*.^45^ did not quantify the maximum of extent of shock experienced by 67215. The weak correlations between Sm/Nd and shock state observed in plagioclase in NWA 2995 (Fig. 6) indicate a potential link between trace-element systematics and shock state. Such a relationship would imply that that weak to moderate shock modification may be sufficient to partially reset/re-equilibrate the Sm-Nd isotope system in plagioclase. We note that due to the typically short duration of impact shock pressure waves (<1 minute^33^), such processes are unlikely to fully reset Sm-Nd ages, which require elevated temperatures at longer time scales to fully re-equilibrate the Sm-Nd (*e.g.,* >0.1 Ma for Nd within 100 μm plagioclase at >1000 °C^48^. No clear relationship is seen between age and peak shock pressures in the samples investigated here. However, the heterogeneous nature of shock (as seen in the range of shock pressures per clast in this study; Table 2), together with the averaging effect of analysing mineral separates, would act to obscure such relationships. Recently, reported young Sm-Nd ages for FAN samples (*e.g*., 4.29 ± 0.06 Ga for 62236^49^) relative to the traditionally expected crystallisation ages for the FANs at (~4.46 Ga^50^) has led some to challenge the long held beliefs that the lunar highlands crystallised following a single event shortly after the formation of the Moon (~200 Ma^50^). In light of this debate, it is important to first establish the shock state of samples prior to interpreting ages derived from the Sm-Nd isotope system, particularly if plagioclase mineral fractions are used to define the isochron.

Due to the generally low REE+Y abundances of plagioclase in lunar FANs, coupled with small clast sizes within breccias, the ^39^Ar-^40^Ar isotope system is commonly used to extract ages for understanding the history of these samples (*e.g*., refs 45, 51–54). Unlike the Sm-Nd system, the Ar-Ar system is very susceptible to impact induced heating resulting in the diffusional behaviour of ^40^Ar at lower temperatures relative to the Sm-Nd system (*e.g*., ref. 55). Ages derived from this system typically represent the age of large thermal re-equilibrium/impact events, generally yielding younger ages relative to the Sm-Nd chronometer (Table 2 summarises Sm-Nd and Ar-Ar ‘ages’ reported for the samples investigated in this study). Nyquist *et al*.^56^ noted that the Ar-Ar ages of Apollo FAN samples generally cluster around the age of the Imbrium and Serenitatis impacts (Apollo FAN average = 4.03 ± 28 Ga). Within the suite of FAN hand samples investigated here, we note that FAN sample 60015 (which was identified as one of the most shocked hand samples within this suite) yields the youngest Ar-Ar plateau age (3.5 ± 0.8 Ga^57^; Table 2). The Ar-Ar system is thought to have low closure temperatures (~200 to 300 °C at 10 °C cooling per Ma, based on terrestrial plagioclase^58^), thus, moderate shock this sample has experienced could be sufficient to modify the Ar-Ar systematics of this sample.

Overall, we highlight that FTIR analysis is a rapid non-destructive method for untangling the shock-history of lunar anorthositic materials. The observation of a weak correlation between some incompatible REE+Y ratios (such as Sm/Nd or La/Y) and moderate to weak shock states in plagioclase from sample NWA 2995, together with the heterogenous nature of shock within single plagioclase grains/clasts suggest that caution should be used when interpreting Sm-Nd isochrons, particularly when using plagioclase mineral fractions to derive sample ages. As also noted by Neal and Draper^60^, inclusion of smaller crystals that have experienced cataclasis and recrystallization may give spurious age results. This study demonstrates the significance of the need to carefully consider the shock state of plagioclase prior to interpreting the emerging picture of geochemical diversity recorded by subsets of anorthosites. This is of particular importance as the extent of geochemical diversity currently identified within lunar anorthosite suites have resulted in new crustal formation hypotheses. Models such as ‘serial magmatism’ (whereby plagioclase crystallised during discrete crystallisation events^14, 62^), and ‘asymmetric flotation’ (whereby the crust on the far side crystallised before the near side^63, 64^) have recently been gaining favour, challenging the ‘classic’ single plagioclase crystallisation event within the lunar magma ocean (*e.g*., ref. 12).

## Methods

The Fourier Transform Infrared (FTIR) analyses of both polished thin-sections and crushed chips (~500 μm to ~1 mm in size) were collected with a Perkin-Elmer Spotlight-400 FTIR spectrometer and an adjoining micro-spectroscopy mapping unit using a cooled Hg-Cd-Te detector. Scans were made from 650 to 4000 cm^−1^ at a 4 cm^−1^ (~15 to 2.5 μm) resolution. Background calibration was conducted using a gold-coated aluminium standard at regular intervals (every 10 to 15 analyses) in order to minimise overall uncertainty related to changes in the thermal background of the surrounding environment. Polished sections were analysed using spot sizes of 25 × 25 µm, integrated over 32 scans. Crushed chips were analysed using the Perkins-Elmer mapping tool integrating a 25 × 25 µm spot size over a ~200 × 200 µm area.

Optical microscope-CL imaging was acquired using a CITL 8200 mk3 ‘cold’ CL system coupled to a transmitted-light microscope; typical conditions were ~10 to 15 kV and a current of ~300 mA. Backscatter Electron (BSE) images were collected using a Phillips FEI XL30 Environmental Scanning Electron Microscope-Field Electron Gun (ESEM-FEG) at the University of Manchester. Beam conditions of 15 keV accelerating voltage and a 20 nA beam current was used. In order to increase the counting statistic for Mg, Fe, Ti and, Mn 64 frames were collected over the areas of interest. Major-elements were collected using a Cameca SX-100 electron microprobe using an accelerating potential of 15 keV, beam current of 10 nA, 1 μm diameter beam, and standard PAP corrections. Counting times were 20 s for Si, Mg, Fe, Na, and Al; 30 s for Ca, Cr, K, and Mn; and 40 s for P. The instrument was calibrated daily using both natural and synthetic standards.

Trace-element analyses were collected on a Cameca ims-4f SIMS at the Edinburgh Ion Microprobe Facility (EIMF). We used a ~10 nA primary beam of negative ^16^O ions accelerated to 15 kV focused to a spot sizes of 25 μm. The acquisition time was 600 s over 10 cycles. Collected isotopes are ^26^Mg, ^47^Ti, ^88^Sr, ^89^Y, ^138^Ba, ^139^La, ^140^Ce, ^141^Pr, ^143^Nd, ^149^Sm, ^151^Eu. Analysed masses were calibrated under the same conditions using NIST-610 glass as an external standard (values compared to those reported in the GeoReM database) and were normalised to Ca as an internal standard (as measured by the Electron microprobe analyser; EMPA). Based on ion counting statistics, the analytical precision (RSD) for the analyses is 2–20% (relative) for the REE+Y, and 1–10% (relative) for the other trace elements.

Full details describing the methods used for estimating plagioclase shock pressures are found in the SOM.

## Electronic supplementary material

Supplementary Information

Table S1
