# Supplementary material for: Assessing the shock state of the lunar highlands: Implications for the petrogenesis and chronology of crustal anorthosites
Source: Sci Rep. 2017 Jul 19;7:5888. doi: 10.1038/s41598-017-06134-x (PMC5517601; doi:10.1038/s41598-017-06134-x)
Supplement: Supplementary file 1 — Supplementary Information [file 41598_2017_6134_MOESM1_ESM.doc]

**Supplementary online materials: Assessing the shock state of the lunar highlands: Implications for the petrogenesis and chronology of crustal anorthosites.**

J. F. Pernet-Fisher, K. H. Joy, D. J. P. Martin, K. L. Donaldson Hanna

*Scientific Reports*

Table S1 is a supplementary excel spreadsheet

**Sample preparation**

*Terrestrial samples:* In order to test the extent to which the ‘unshocked’ FTIR spectra reported by Johnson et al. (2002) truly represent unshocked crystalline plagioclase, we analysed the FTIR spectra of plagioclase from the terrestrial Stillwater and Shawmere intrusive anorthosite complexes and of Miyake Jima megacrysts collected from the 1874 ash flow. We acquired spectra from both polished thin-sections and unpolished chips in order to directly compare with the spectra of Stillwater chips reported by Johnson et al. (2002). Further details are in the next section. Sample chips were prepared by gently crushing plagioclase to a ~1 to 2 mm size fraction using an agate mortar and pestle.

**Mineral shock state classification**

The characterisation of the shock state of minerals have traditionally been classified using polished thin-sections relying on systematic changes in mineral behaviour under crossed polarized light, and more recently using Raman spectroscopy (Stöffler et al., 1991; Rubin et al., 1997; Fritz et al., 2017). This classification scheme, divided into 6 shock states, the crossed-polarised light behaviour of plagioclase, and the equivalent shock pressures under this scheme are summarised **Table S1**. The estimated shock pressure based on the FTIR analyses are presented in **Table 2** alongside the Stöffler et al. (1991) and Rubin et al. (1997) scheme for comparison.

**Table S2**: Optical shock classification scheme.

| Shock stage | Optical properties | Shock pressure (GPa) |
| --- | --- | --- |
| Unshocked  S1 | Sharp optical extinction,  irregular fractures | <5 |
| Very weakly shocked  S2 | Undulatory extinction, irregular fractures | 5 to 10 |
| Weakly shocked  S3 | Undulatory extinction | 10 to 15 |
| Moderately shocked  S4 | Undulatory extinction, partially isotropic, planar deformation features | 15 to 30 |
| Strongly shocked  S5 | Maskelynite (fully isotropic) | > 30 |
| Very strongly shocked  S6 | Shocked melted (normal glass) | >45 |

**Quantifying maximum impact shock pressures.**

In order to quantify the maximum impact shock pressure experienced by plagioclase for a range of planetary materials, a variety of methods have been utilized within the current literature, including optical microscopy observations (Stöffler et al., 1991; Rubin et al. (1997), Raman spectroscopy (*e.g.,* Fritz et al., 2005; Fernandes et al., 2010), and FTIR (*e.g.,* Johnson et al., 2002; Johnson, 2012). Here we employ FTIR methods, which rely on systematic changes in the FTIR reflectance spectra with increasing shock pressure. Specifically, the strength of a number of characteristic plagioclase spectral features (such as the 790 cm^-1^ to 900 cm^-1^ and 1075 cm^-1^ to 1150 cm^-1^ transparency feature and reststrahlen bands, respectively; Johnson et al., 2002) have been shown to systematically decrease with increasing shock pressure. Using a suite of experimentally shocked plagioclase chips, this relationship was used by Johnson et al. (2002) and Johnson, (2012) to estimate shock pressures for ‘unknown’ plagioclase crystals of interest.

For this study, we estimate shock by use the experimentally shocked high-Ca plagiolcase chips (An_82_; from the Stillwater Complex, Montana, USA) reported by Johnson et al. (2002) and apply new measurements of an unshocked plagioclase crystals (An_97_) from the Miyake-Jima 1874 ash flow (Japan). In order to assess the effects of shock, we track the decrease in the absolute reflectance at 1126 cm^-1^ (calculated as the band depth at 1126 cm^-1^ using continuum-removed tie points between 1075 cm^-1^ and 1200 cm^-1^) with increasing shock presure. (Continuum removal normalises spectra in order to directly compare individual absorption/reflectance features of spectra from a common baseline). This band takes advantage of the fact that the reststrahlen bands between ~1150 and ~1050 cm^-1^ are lost during the formation of glass, irrespective of plagioclase chemistry. Furthermore, at this specific band position there are no differences that result from crystal orientation effects. Indeed, for polished samples, depending on which crystal orientation is investigated, some of the characteristic absorption features used to estimate shock pressure display significantly different band maxima/minima and intensities.


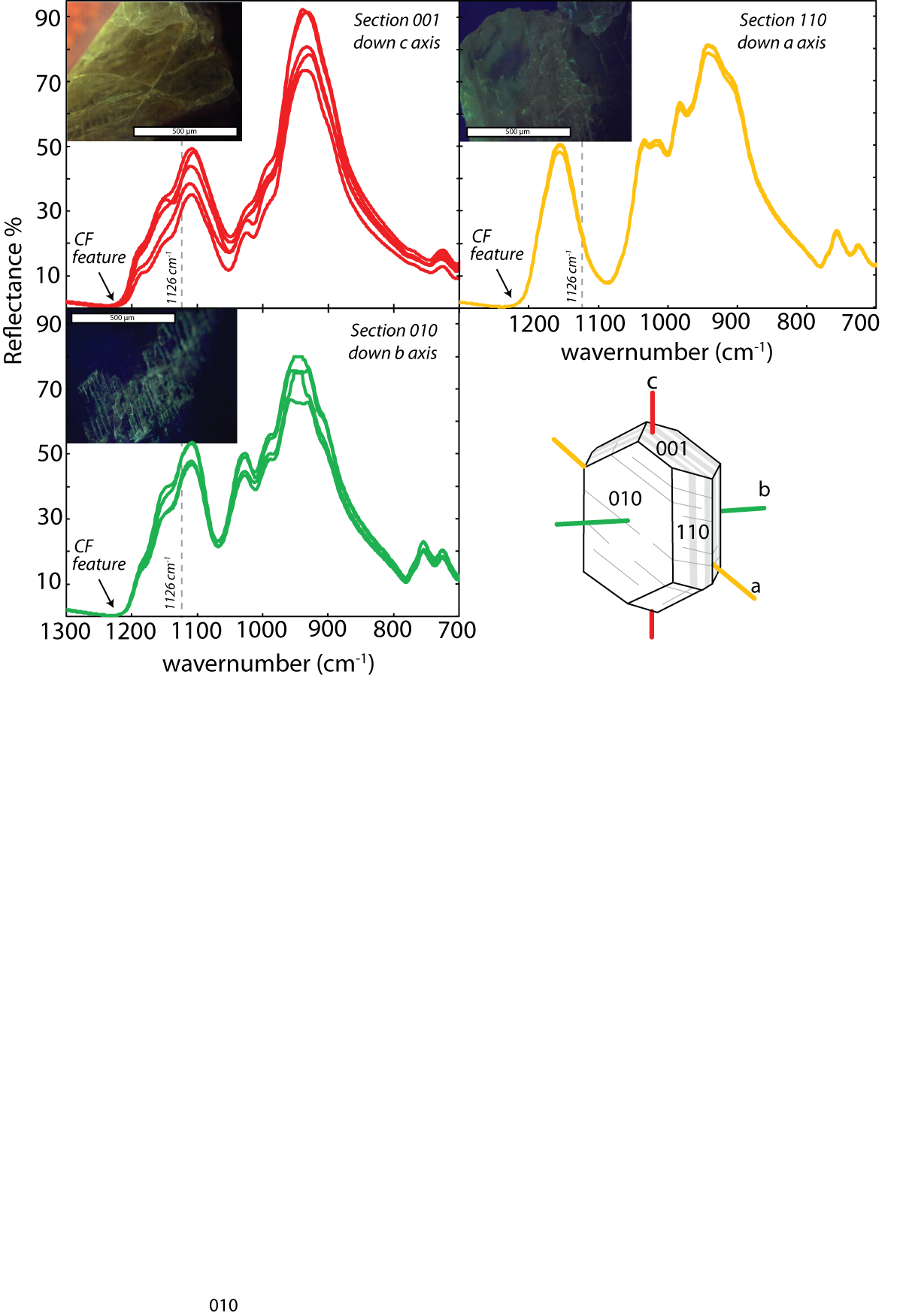


***Figure S1:*** *Reflectance (%) spectra of polished blocks of known crystal orientations of the Miyake-Jima plagioclase. In each panel, the OM-CL image of the analysed mineral grain is included.*

The well-formed tabular nature of the Miyake-Jima plagioclase is such that orientation effects on the FTIR reflectance spectra can be investigated. **Figure S1** displays the FTIR spectra for the “001” (red), “110” (yellow), and “010” (green) crystal orientation planes, with associated CL-images of the sample studied. Despite the significant differences in FTIR spectra, CL-imaging is not sensitive to orientation effects (**Figure S1)**. **Figure S2** displays the average continuum removed (from 1075 cm^-1^ to 1200 cm^-1^) spectra for each crystal orientation of the Miyake-Jima plagioclase (transparent fields around spectra represent 1σ error based on repeat analyses on the same crystal face; *i.e.,* reflecting the external reproducibility). The continuum removed tie points were optimised to ensure all 3 orientations intersect each other at same band depth, in this case at the 1126 cm^-1^ wavelength. This wavelength was selected as at this position, as each orientation has band depths within error of the external reproducibility.


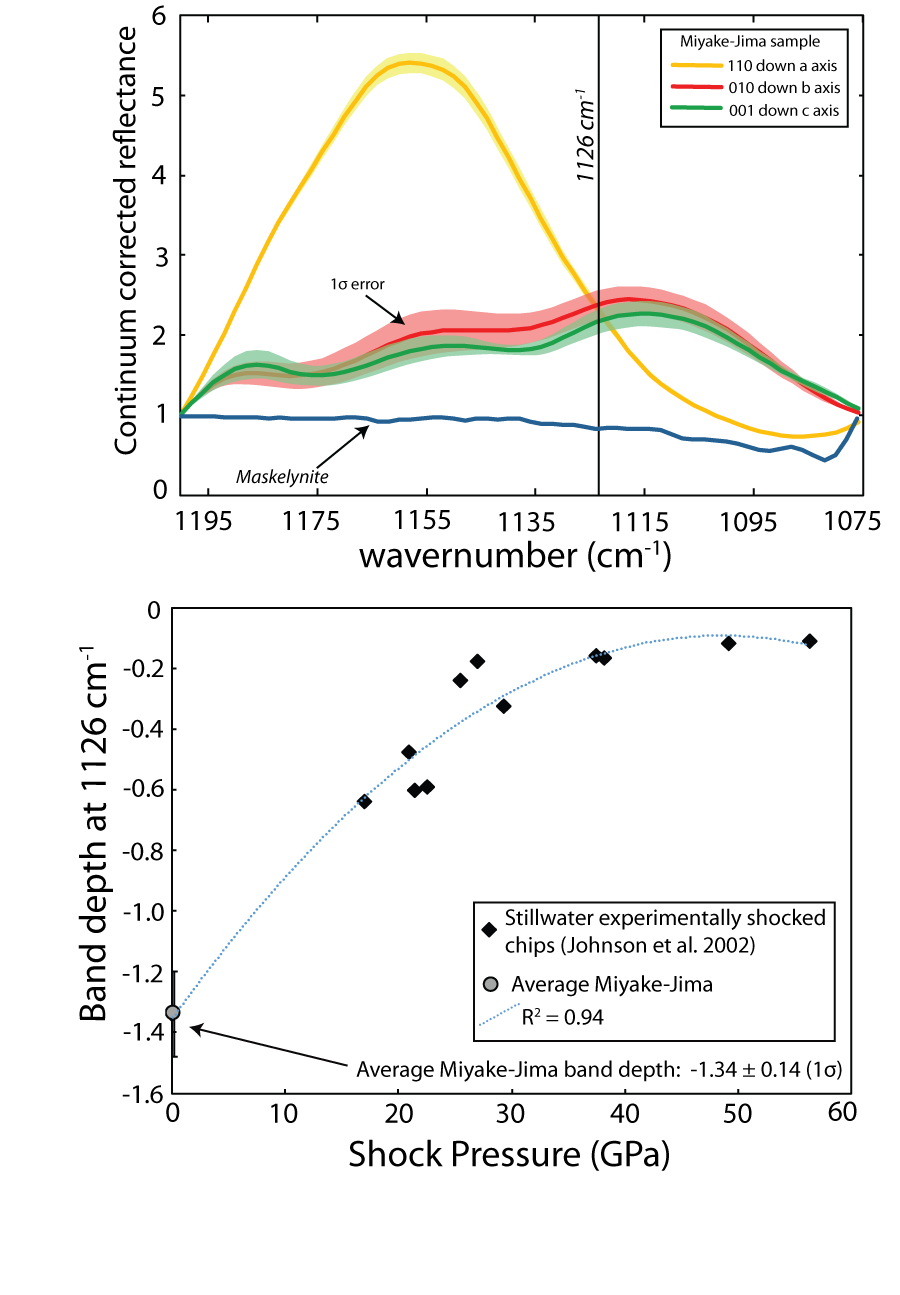


***Figure S2:*** *Average continuum corrected reflectance (%) spectra between 1075 cm^-1^ and 1200 cm^-1^ for 3 orientations (“110”, “010”, and “001”) of polished Miyake-Jima plagioclase crystals. Transparent fields around average values reflect 1 σ error based on repeat analyses of the same crystal face. To illustrate the disappearance of this band feature with glass formation, the spectrum of a lunar maskelynite grain of similar major element composition (from Apollo sample 61175,108) is also plotted for context.*

At the 1126 cm^-1^ band position, the experimentally shock plagioclase reported by Johnson et al. (2002) displays a good correlation between band depth vs. shock pressure (**Fig. S3**) although we note that a non-linear fit yields a better fit (r^2^ = 0.94) than a linear regression (as suggested by Johnson et al., 2002). The experimentally shocked plagioclase (black diamonds) fall within relatively high shock pressures (> 17 GPa); thus, regressing the correlation observed to lower (< 17 GPa) pressures relies on the robust characterisation of unshocked (i.e., 0 GPa) plagioclase.

***
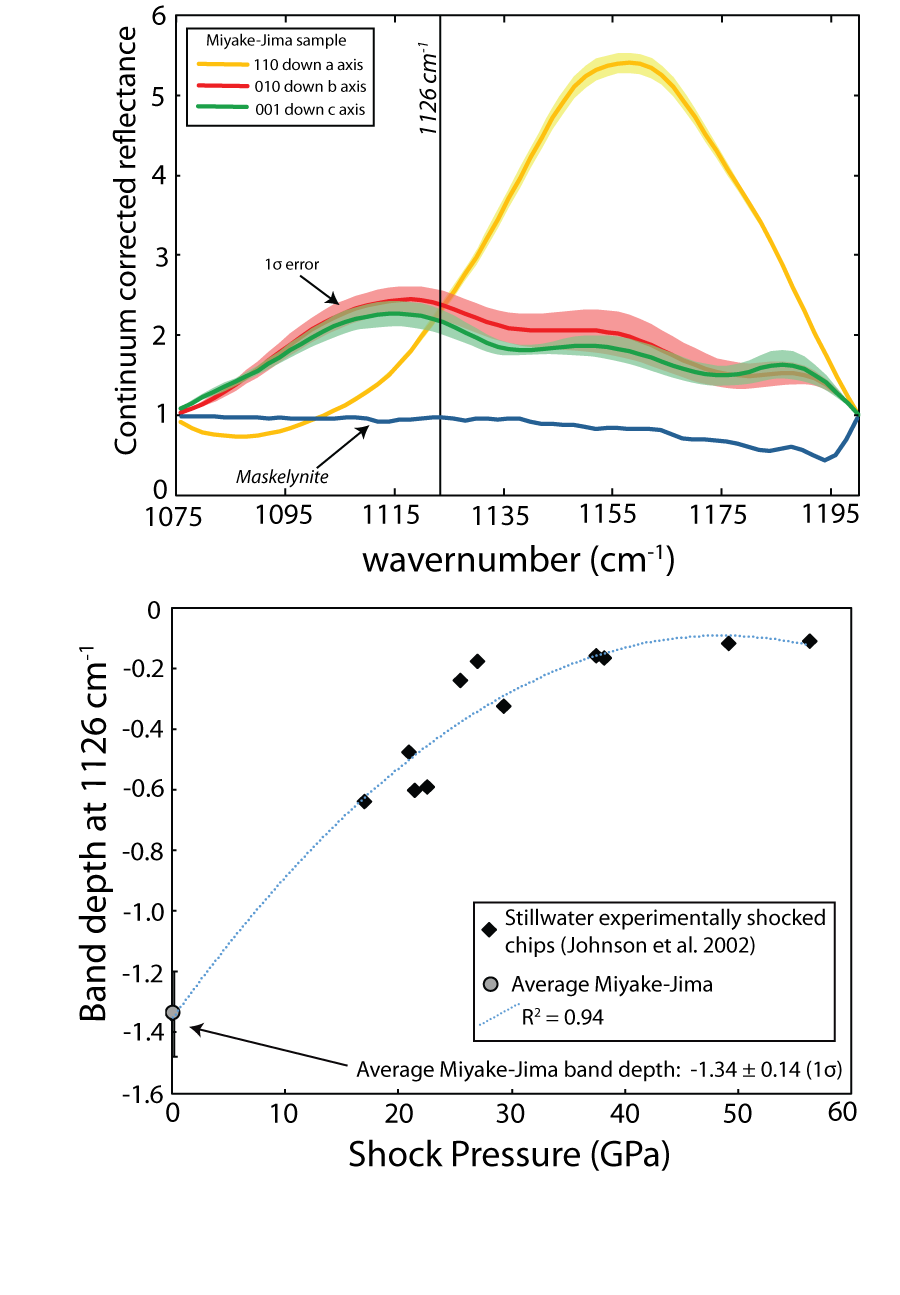
***

***Figure S3:*** *Band depth at 1126 cm^-1^ (calculated using a continuum between 1075 cm^-1^ and 1200 cm^-1^) vs. shock pressure for experimentally shocked plagioclase chips (from Johnson et al., 2002) and average spectra for Miyake-Jima plagioclase. Non-linear (quadratic function) correlation coefficient is also shown.*

To investigate potential spectral differences between polished sections and chips, we analysed both crushed chips (~1 to 2 mm in size) and polished thin-sections of the Miyake-Jima and Stillwater samples (sample STL-100, McCallum et al., 1981). The analyses of crushed chips enables the spectra from these samples to be directly compared with the crushed chip spectra reported by Johnson et al., (2002) who reported the FTIR spectra for chips ~2 mm in size. Despite absolute differences in %R, the relative band positions of both polished sections and chips have overlapping band ratios displayed in **Fig. S4**. Despite this, the Stillwater complex is known to have undergone both regional and contact thermal metamorphism following their emplacement (e.g., Page, 1977; Labotka and Kath, 2001), thus, we suggest that the ‘unshocked’ Stillwater sample reported by Johnson et al. (2002) does not actually reflect unshocked crystalline plagioclase. This is shown by FTIR the loss of distinct absorption bands (such the 950 cm^-1^ to 1150 cm^-1^ feature) with respect to the
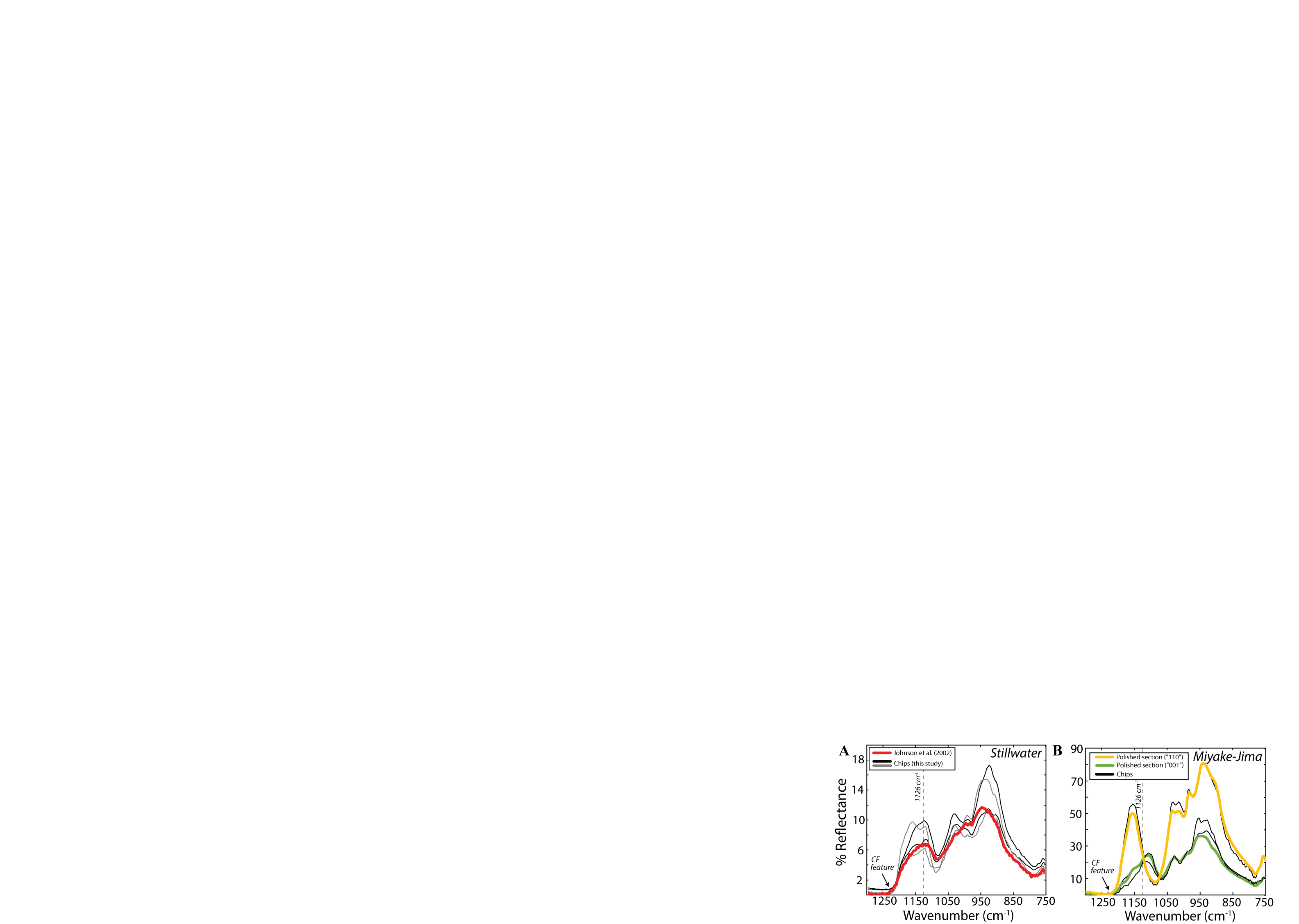
volcanic Miyake-Jima plagioclase, which in turn will yield spurious shock pressures.

***Figure S4****:* ***A.*** *Reflectance FTIR spectra for crushed Stillwater chips (1 – 2 mm in size) from this study (black and grey lines) vs. the unshocked ~2 mm chips reported by Johnson et al. (2002) (red lines; to directly compare the reported emissivity spectra of Johnson et al. (2002), we have plots 1-emissivity). Samples display differences in absolute % Reflectance, thus, in order to directly compare relative band positions our samples (grey lines) with the experimental data of Johnson et al (2002), the % reflectance values for our samples have been divided by a factor of 5.* ***B.*** *FTIR spectra for polished sections (yellow and green lines) and crushed 1-2 mm chips (black line) of Miyake-Jima plagioclase. As noted above, to better compare the relative band differences, the spectra for “001” orientation (green line) has been divided by 2.*

Instead, we prefer to use the Miyake-Jima plagioclase to ‘anchor’ the best-fit lines of the experimentally shock plagioclase to 0 GPa (**Fig. S3**). The external reproducibility (based on repeat measurements of single crystal faces of Miyake-Jima during the analytical campaign) represent the largest source of error (see 1σ field around spectra in **Fig. S2**). When this error is propagated to the best fit trend in **Fig. S3**, the typical errors for the pressure calculations is ± 3 GPa. All Miyake-Jima and Stillwater analyses collected over the course of this study are also presented in **Table S1**.

***Figure S5:*** *CL emission images coupled with qualitative element concentration distribution maps for Mg, Mn, and Ti for select plagioclase crystals that display variations in CL emission colour or intensity within a single grain. Crystal edges are shown are white-dashes are shown for reference.*

*
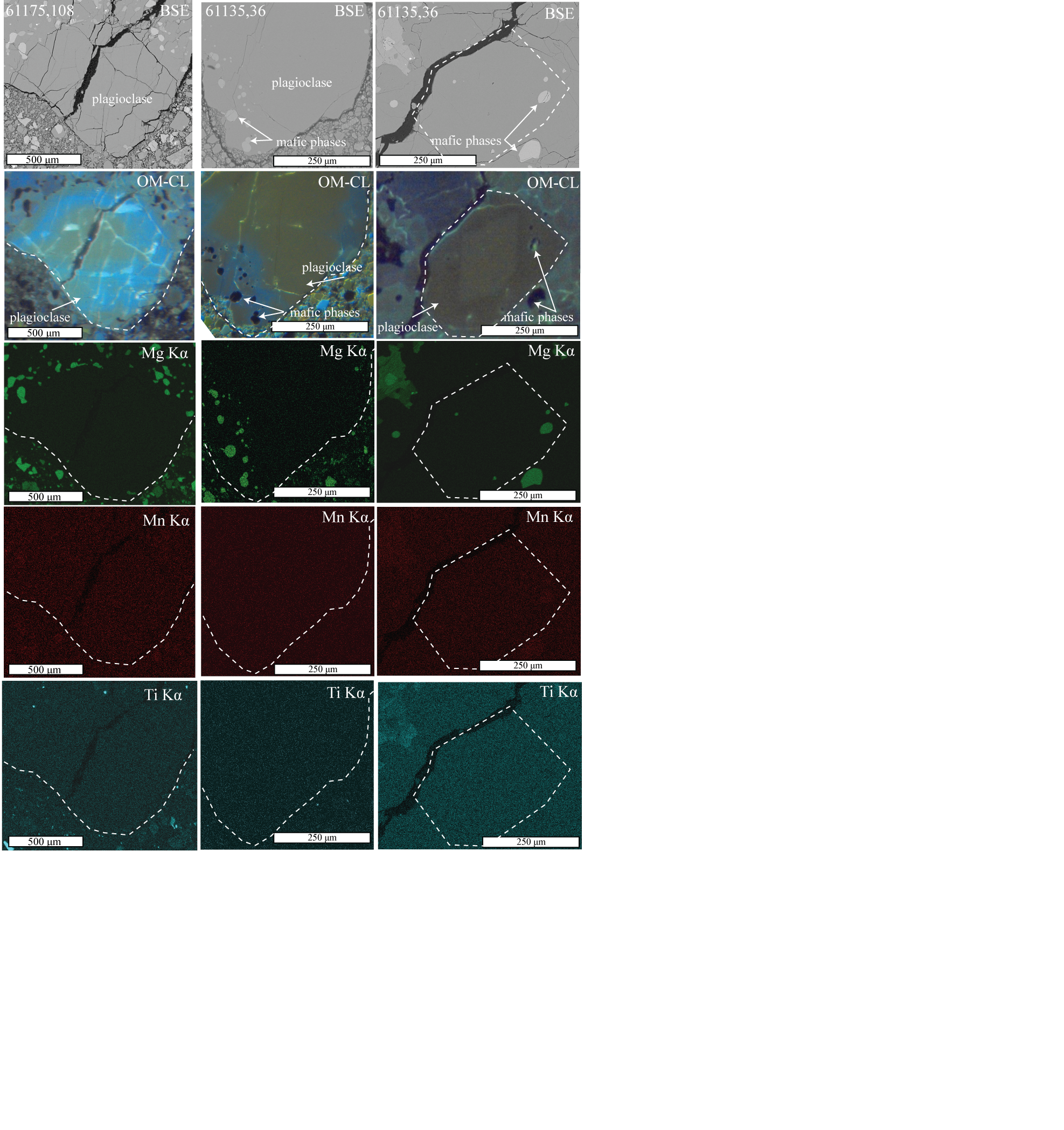
*

***Figure S6:*** *CL and BSE images for all FAN clasts/mineral fragments within the Apollo 16 breccia investigated.*

*
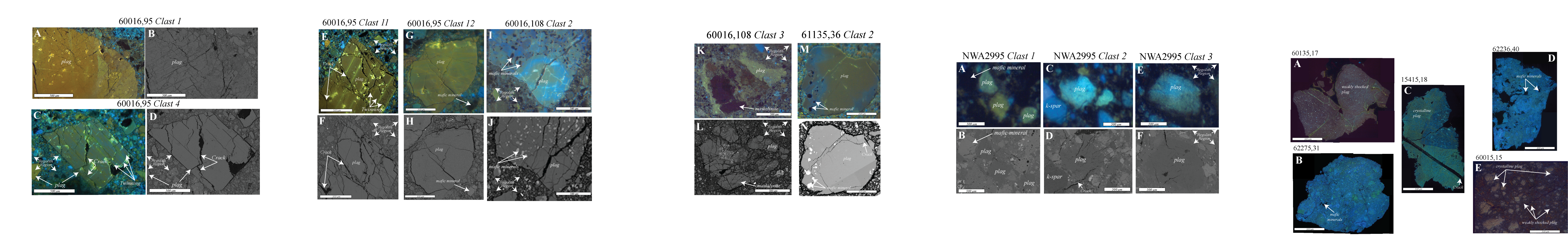
*

***
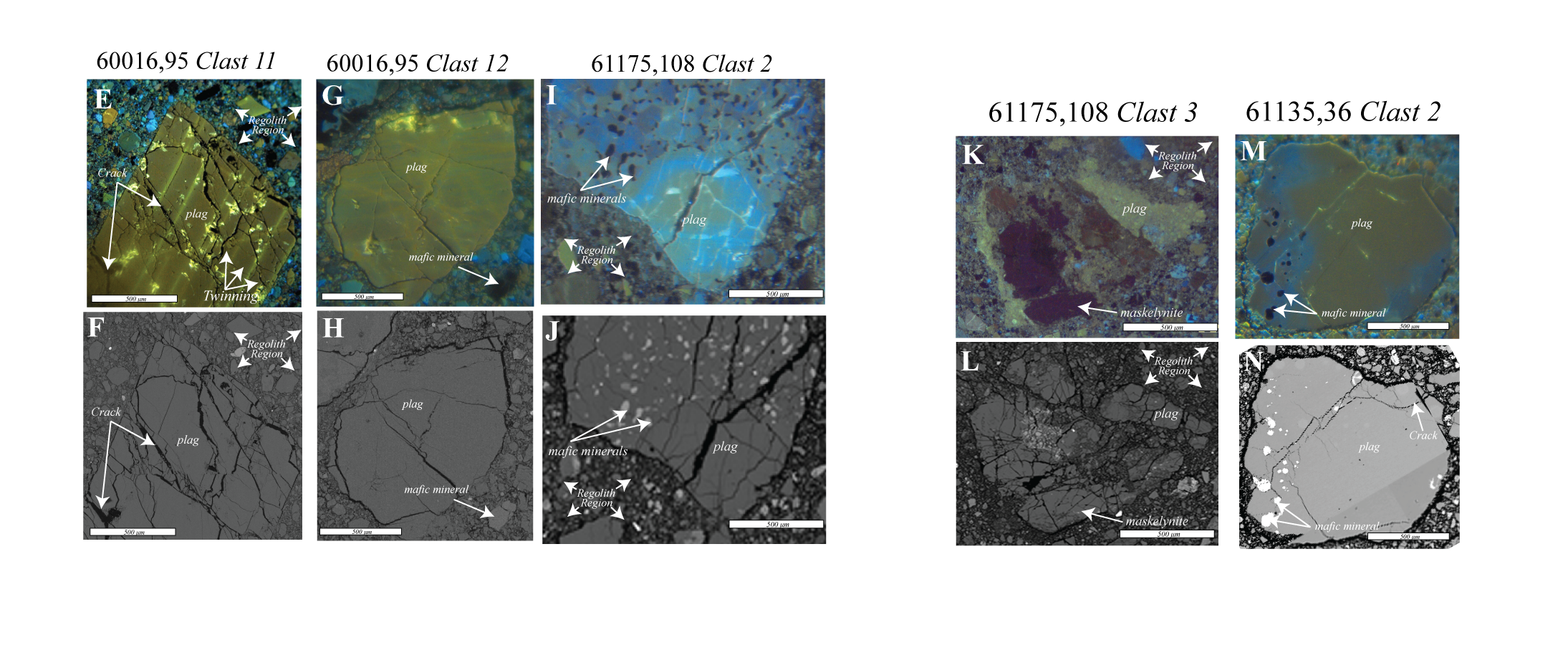
***

***
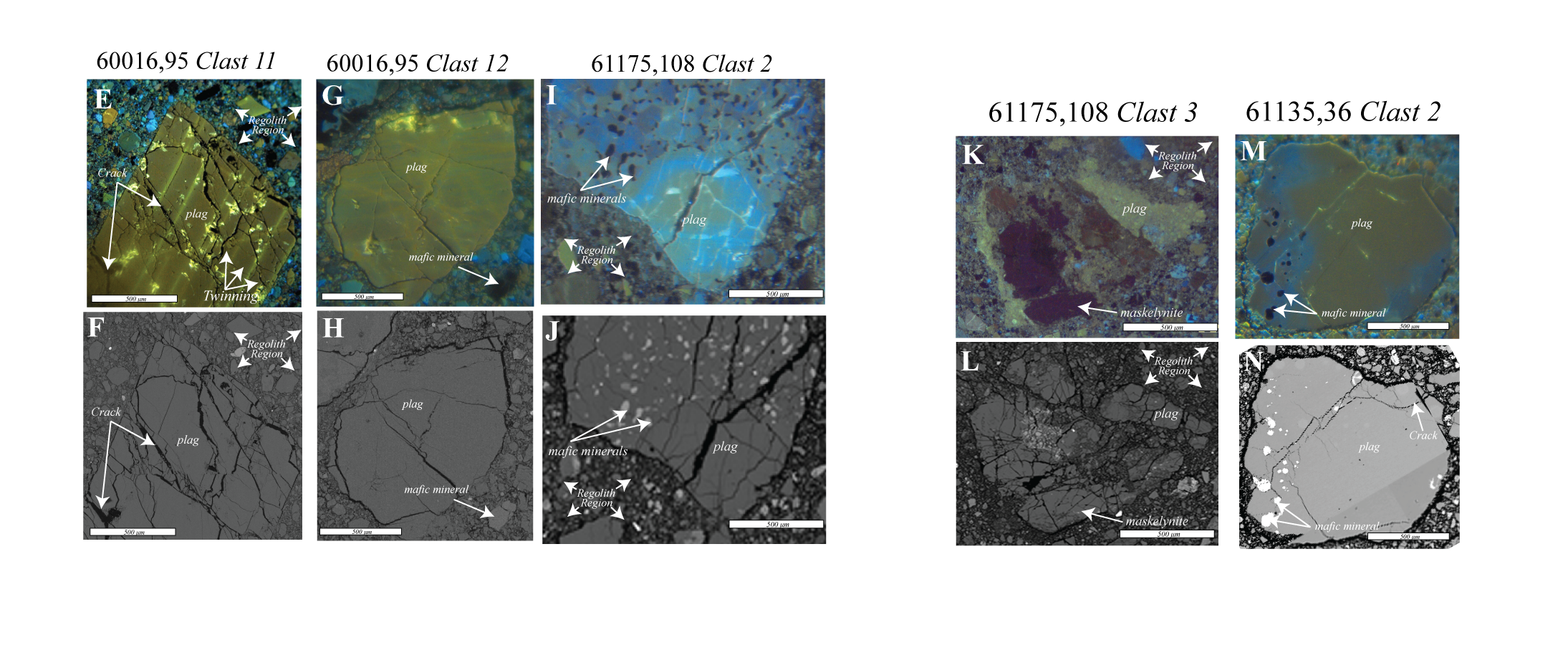
***

***Figure S7:*** *CL and BSE images for all FAN clasts/mineral fragments lunar meteorite NWA 2995.*

*
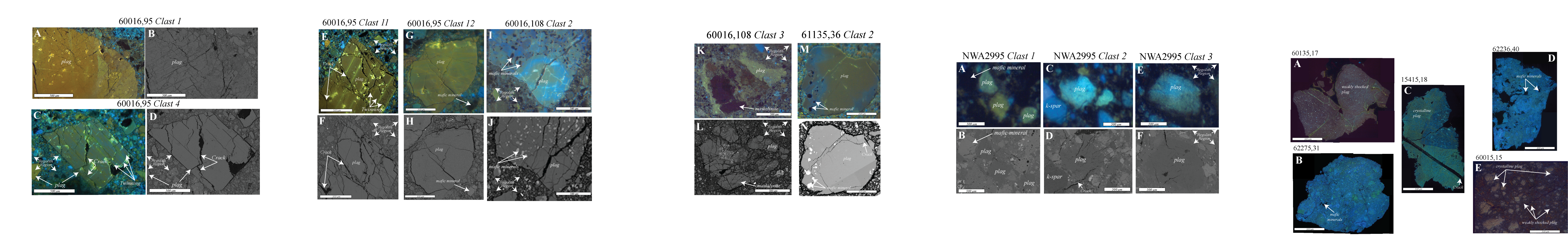
*

***Figure S8:*** *CL images for the Apollo FAN hand specimen samples investigated here.*

*
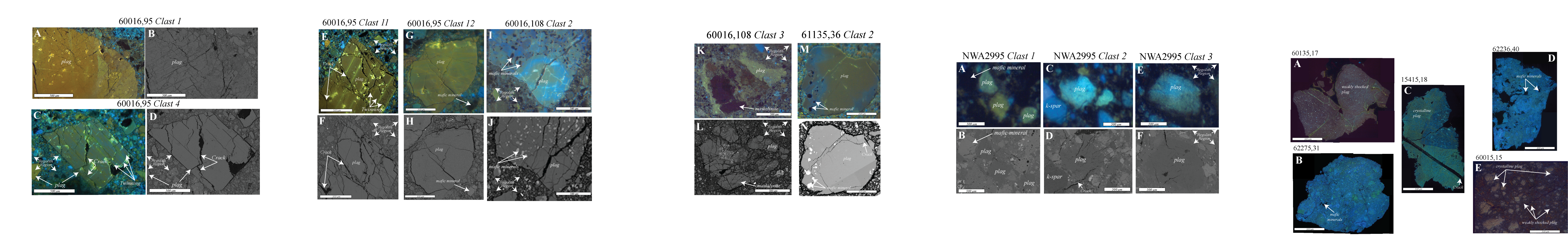
*

***Figure S9:*** *Photomirograph under plain polarised light and crossed poliazed light of clast 3 in sample 61175,108. Isotropic nature of plagiolcase in clast suggests it has been transformed into its highpressure psudomorph maskeynite (see main text for details).*

*
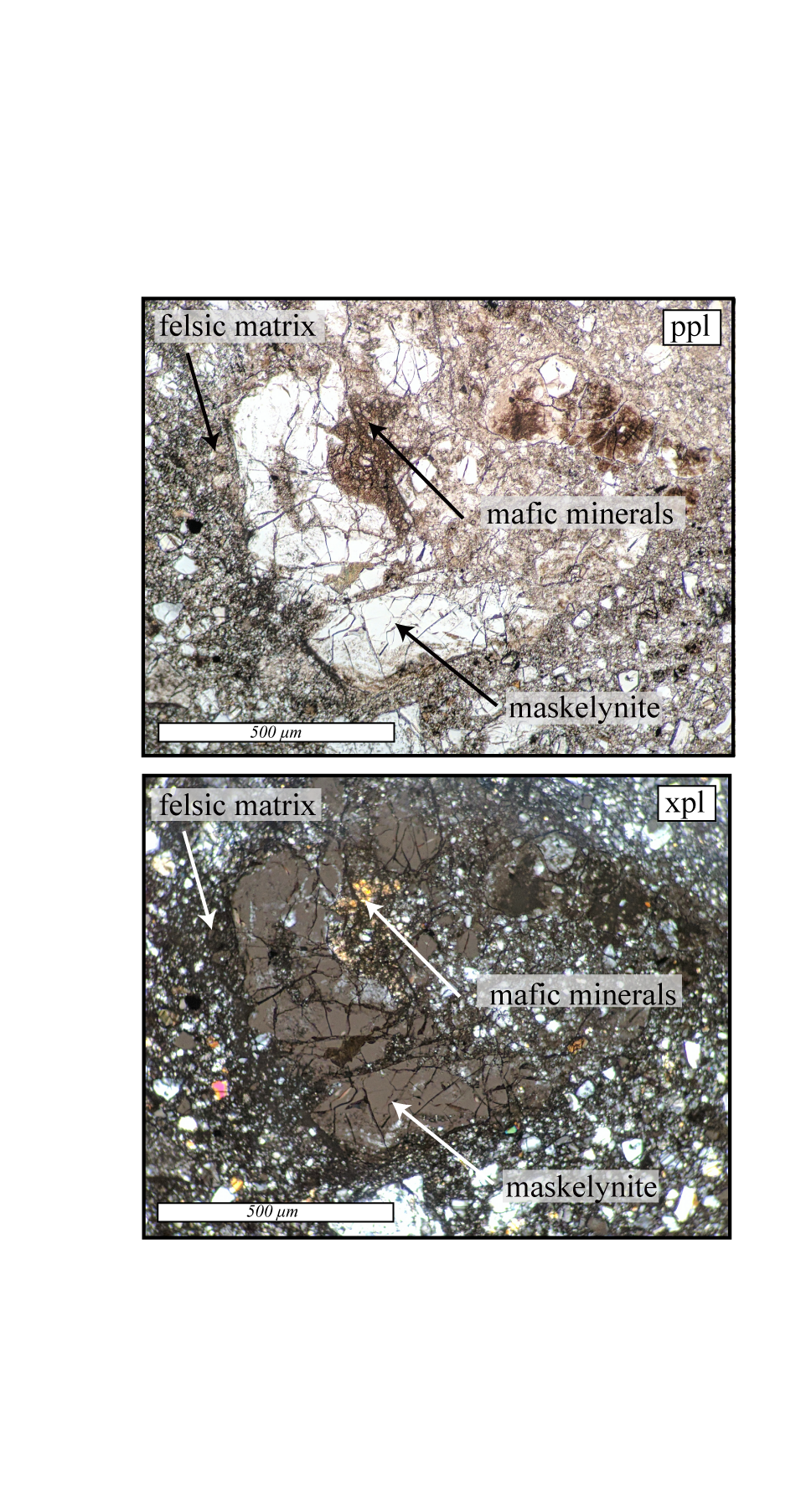
*

**Supplementary References cited:**

Fernandes, V. A., Fritz, J., Weiss, B. P., Garrick‐Bethell, I., & Shuster, D. L. (2013). The bombardment history of the Moon as recorded by 40Ar‐39Ar chronology. Meteoritics & Planetary Science, 48(2), 241-269.

Fritz, J., Greshake, A., & Stöffler, D. (2005). Micro-Raman spectroscopy of plagioclase and maskelynite in Martian meteorites: Evidence of progressive shock metamorphism. Antarctic meteorite research, 18, 96.

Fritz, J., Greshake, A., & Fernandes, V. A. (2017). Revising the shock classification of meteorites. Meteoritics & Planetary Science.

Johnson, J. R. (2012). Thermal infrared spectra of experimentally shocked andesine anorthosite. Icarus, 221, 359-364.

Johnson, J. R., Hörz, F., Lucey, P. G., & Christensen, P. R. (2002). Thermal infrared spectroscopy of experimentally shocked anorthosite and pyroxenite: Implications for remote sensing of Mars. Journal of Geophysical Research: Planets, 107(E10).

Labotka, T. C., & Kath, R. L. 2001. Petrogenesis of the contact-metamorphic rocks beneath the Stillwater Complex, Montana. Geological Society of America Bulletin, 113, 1312- 1323.

McCallum, I. S., Raedeke, L. D., & Wiesmann, H. 1981. Fractionation Trends in the Lunar Crust and the Stillwater Complex Trace Element Data. In Lunar and Planetary Science Conference (Vol. 12, pp. 676-678).

Page, N. J., 1977, Stillwater Complex, Montana: Rock succession, Metamorphism and structure of the complex and adjacent rocks, Geological Survey professional paper, 999.

Rubin, A. E., Scott, E. R., & Keil, K. 1997. Shock metamorphism of enstatite chondrites. Geochimica et Cosmochimica Acta, 61, 847-858.

Stöffler, D., Ryder, G., Ivanov, B. A., Artemieva, N. A., Cintala, M. J., & Grieve, R. A. 2006. Cratering history and lunar chronology. Reviews in Mineralogy and Geochemistry, 60, 519-596.
